# Review of "Keys to the Nematode Parasites of Vertebrates. Supplementary Volume" by Lynda M. Gibbons

**DOI:** 10.1186/1756-3305-3-9

**Published:** 2010-02-18

**Authors:** František Moravec

**Affiliations:** 1 Institute of Parasitology, Biology Centre of the Academy of Sciences of the Czech Republic, Branišovská 31, 370 05 České Budějovice, Czech Republic

## Abstract

Book review of "Keys to the Nematode Parasites of Vertebrates. Supplementary Volume" by Lynda M. Gibbons

## Review

For many years, the "CIH Keys to the Nematode Parasites of Vertebrates", authored by Anderson, Chabaud and Willmott (eds.) in ten parts between 1974 and 1983, served as a main and absolutely essential working tool for the generic identification of nematodes parasitic in vertebrates. However, a number of taxa were deliberately omitted by the editors, because their validity was doubtful and the type species of dubious status (these were later listed in the paper of Spencer Jones & Gibson [1]). Due to popular demand, a re-publicatin of the refreshed Keys with reordered superfamilies in a single, archival volume appeared last year and was reviewed in this journal.

However, it is clear that classifications are changing as new taxa are described and new techniques are developed, especially in such a large group as parasitic nematodes of vertebrates, so that any comprehensive monograph treating these parasites as a whole becomes quickly out of date. It is 36 years since the original publication of the Keys and, therefore, it can be highly appreciated that a supplementary volume is now available, soon after the recent re-issue of the archival volume of the Keys. Of course, it is not easy to make any supplements to the classification system of these parasitic organisms after so many years, because the views of authors concerning the validity of various taxa and their position in the system may differ considerably. This very difficult work was undetaken by Lynda Gibbons, a well-known world expert for nematode parasites of vertebrates, especially as to their morphology and taxonomy. She coped with this task very well and produced an excellent book, which, along with the archival volume of the Keys, will undoubtedly serve for years as an indispensable tool for the identification of these parasites.

The text of the volume is divided into 10 chapters. After the short Introduction and Acknowledgements (3 pages), the following 6 chapters (371 pages) deal with individual nematode orders Enoplida, Rhabditida, Strongylida, Oxyurida, Ascaridida and Spirurida and form the core of the book, covering 479 genera (of which about 300 were not previously reported in the Keys) and 78 subgenera belonging to 76 families and 94 subfamilies. As the author mentions, the aim of this compilation volume of new, revised and redescribed taxa was not to make a major revision of nematode classification, but to draw attention to new taxa published after the Keys were completed and to include some of the older taxa omitted in the original.

Supplements in each of the chapters treating individual nematode orders are arranged in accordance with the classification used in the original Keys and, where new suborders or superfamilies have been proposed since their publication, these are reviewed under the relevant taxa. Each of the taxa listed has a brief diagnosis based on the original reference in the Bibliography, unless otherwise stated within the quare brackets. The diagnoses of some taxa previously present in the Keys are supplemented to include additional descriptions or revised classification. However, as the author remarks, both the supplemented diagnoses and the taxa not previously included should be consulted with the original references. Comments on the history and any changes in status of any of the taxa can be then found between square brackets. The book is provided with Bibliography (31 pages) and Index (12 pages) including the names of all taxa, except for those of species. The text of this publication contains 325 plates of line drawings of generally good quality, illustrating the main taxonomic features.

Without doubt, it is very difficult to compile such a comprehensive publication of this type and I admire the author's efforts to realize it. I am not sure if it was a good decision to include some evidently doubtful taxa, which were omitted in the previously published Keys, such as, e.g., the genera *Neocylicostrongylus *Arya and Johnson, 1977, *Kheraia *Rautela and Malhotra, 1985, *Neostrongyloides *Rathore and Nama, 1985 or *Cylicostrongylus *Yamaguti, 1961 (the type species of the latter was apparently based on an erroneously described, incomplete body of *Cucullanus truttae *- [2]), because these data may be misleading and may complicate the identification of nematodes. The capillariid genus *Hepatospina *Thieme, 1961 (reported also by Spencer Jones & Gibson [1]) is in fact invalid, because it has never been formally established, *Batrachocamallanus *Jackson and Tinsley, 1995 was later synonymized with *Procamallanus *Baylis, 1923 [3], and *Ichthyonema *Diesing, 1861 is mis-spelled (page 285). Sometimes the use of some names of the genera and other taxa is not in accordance with the presently valid International Code of Zoological Nomenclature (Fourth Edition), which concerns the principle of coordination in genus-group and family-group nominal taxa, i.e., that the authorship is unaffected by changes in rank. For example, the reported Trichuridae (Ransom, 1911) Railliet, 1915 should be correctly cited as Trichuridae Ransom, 1911 or the reported subgenus *Pseudocapillaria *(Freitas, 1959 genus) should be cited as the subgenus *Pseudocapillaria *Freitas, 1959.

Nevertheless, these small shortcomings are negligible and the book as a whole is very well prepared, provides a huge amount of information and, at present, it represents, along with the archival volume of the Keys, the only important publication enabling the generic identification of all nematode parasites of vertebrates. Thanks to this publication and to recently published "Keys to Cestode Parasites of Vertebrates" [4] and the three-volume "Keys to the Trematoda" [5-7], now there are at our disposal keys to genera of nearly all groups of helminths parasitizing vertebrates.

As it has already been stated in the review to the archival volume of the Keys, there is a persistent demand and need for means to identify parasitic nematodes as well as a resurgent interest in the origins and evolution of parasitism within the phylum. Therefore, the publication of this supplementary volume of the Keys is very important and this excellent book, together with the archival volume of the Keys, no doubt represents one of the basic works on parasitic nematodes of vertebrates, which may also serve as a basis for subsequent revisions of taxonomy as well as for studies on the biology, ecology, zoogeography, etc. of these parasites. This will certainly be appreciated as an important source of information and an indispensable aid for the determination of nematodes not only by parasitologists, but also interested physicians, veterinarians, zoologists, wildlife and fisheries biologists, university students and others. The author, editors and the publishers are to be congratulated on this publication.

## Competing interests

The author declares that they have no competing interests.
